# Imported Skin Diseases

**DOI:** 10.3201/eid1406.080223

**Published:** 2008-06

**Authors:** Kenneth Dardick

**Affiliations:** * Connecticut Travel Medicine, Storrs Mansfield, Connecticut, USA

The foreword to Imported Skin Diseases states, “This book was written and illustrated for the health professional in order to help in the diagnosis and management of patients with diseases acquired in another, often tropical, environment.” The book identifies an important clinical need for practitioners whose patients are traveling to an ever greater degree to more distant and exotic locales.

This book is neither an encyclopedic compendium of all tropical skin diseases nor a simple handbook for the house officer or medical student. The breadth of topics is broad, but some topics are discussed in great depth with good authority. The photographic images are clear and well chosen. Chapters that stand out include those on Pigmentary Disorders in Black Skin by J.P.W. van der Veen, M.F.E. Leenarts, and W.W. Westerhof; Coloured versus White Skin by B. Naafs; Buruli Ulcer by F. Portaels and W.M. Meyers; Tungiasis by H. Feldmeier and J. Heukelbach; Myiasis by D.A. Burns; and Beetle Dermatitis by P. Schmid-Grendelmeier and S. Haug.

The chapter Persistent Insect Bites by C.L.M. van Hees assumes that all mosquitoes are night biters. The author states that one can prevent mosquito bites by “avoiding being outside from dusk till dawn.” But this message misses the point because anopheline and other species bite from dusk to dawn, and *Aedes* and other species are day biters. This advice is really intended to help avoid contracting malaria, not being bitten.

At times, the writing is puzzling. For example, the chapter, Fever and Rash, by H.G. Schipper and P.A. Kager discusses loiasis and indicates that “fever is absent.” It is unclear why a chapter on fever would include a disease that has no fever. Loiasis is more fully covered in the later chapter, Onchocerciasis/Filariasis, by M. Murdoch, so its discussion in Fever and Rash is superfluous and out of place.

Many of the chapters were written by authorities whose first language was probably not English. These chapters could have been edited more carefully because grammatical errors interfere with comprehension. The same applies to the book’s typographical errors, which are too frequent to count.

Imported Skin Diseases is organized primarily by diagnosis rather than by syndrome. The disease descriptions are generally complete, with adequate sections on diagnosis and treatment. I discovered at the back of the book, almost by accident, some rather skimpy tables organized by syndrome (fever and rash, ulceration, eschar). I would have preferred this approach to be covered in greater detail because it would be much more useful to the Western practitioner who is confronted with a patient returning from the tropics with an undiagnosed skin disorder. For example, I have a patient in my office with an eschar. The patient has returned from a trip to Africa. What are the diagnostic considerations? Or, a patient has a rash and fever and has traveled widely throughout Asia; how can I approach a proper diagnosis?

As the book is written it could be useful as preparation for the Diploma in Tropical Medicine and Hygiene, certification examination of the International Society of Travel Medicine or American Society of Tropical Medicine and Hygiene, or perhaps as a primer for a physician who will travel to the tropics to practice medicine.

